# Editorial Comment to Syndrome of inappropriate antidiuretic hormone secretion as a side effect of chemotherapy for testicular cancer: A case report

**DOI:** 10.1002/iju5.12125

**Published:** 2019-10-21

**Authors:** Takeshi Yuasa

**Affiliations:** ^1^ Department of Urology Cancer Institute Hospital Japanese Foundation for Cancer Research Ariake Tokyo Japan

Metastatic germ cell cancer (GCC) is a chemotherapy‐sensitive and potentially curable disease. Patients with metastatic GCC therefore require hard chemotherapeutic treatment. The bleomycin, etoposide, cisplatin (BEP) regimen, which consists of cisplatin (20 mg/m^2^, days 1–5), etoposide (100 mg/m^2^, days 1–5), and weekly bleomycin (30 mg, days 1, 8, and 15), is the standard regimen for induction chemotherapy in these patients. The current guidelines recommend the three cycles of BEP for patients with favorable prognosis and four for those with intermediate or poor prognosis according to the International Germ Cell Cancer Collaborative Group (IGCCCG) classification.^1^ This chemotherapeutic regimen is intense and often causes severe adverse events such as febrile neutropenia. Another serious adverse event associated with cisplatin‐based hard chemotherapeutic regimens such as BEP is syndrome of inappropriate antidiuretic hormone secretion (SIADH).^2^

In this issue of *IJU Case Report*s, Maeda *et al*. reported the case of a metastatic GCC patient who developed SIADH in the first cycle of BEP induction chemotherapy.^3^ In previously reported cases of cisplatin‐related SIADH, cisplatin has been replaced with another drug or chemotherapy has been discontinued; Maeda *et al*., however, continued to administer BEP chemotherapy and successfully treated this patient.^3^ They demonstrated that the threat of SIADH as a severe adverse event can be overcome through the careful monitoring of sodium levels and appropriate mineral recruitment.^3^

I would like to send a message to readers as well as to the authors. Although the BEP regimen is the gold standard for induction chemotherapy in patients with metastatic GCC, we need not persist with the BEP regimen when complications arise. We have to remember that there are suitable alternative regimens in which no agent is administered on day 8 or day 15. The BEP regimen includes a weekly bleomycin administration, which sometimes needs to be avoided due to severe adverse events such as febrile neutropenia around day 15. In fact, Maeda *et al*. noted that the patient in their present report could not safely receive bleomycin on day 15 in the first BEP cycle because of a severe adverse event.^3^ Skipping scheduled doses in this way reduces the relative dose intensity: removing the bleomycin from the BEP regimen makes it comparable to the etoposide/cisplatin (EP) regimen used to treat patients with favorable‐risk metastatic seminoma, and it has been reported that four cycles of the EP regimen have an efficacy equivalent to that of three cycles of the BEP regimen.^4^ In addition, when the BEP regimen cannot be conducted according to the prescribed dosage schedule, an alternative is the cisplatin, etoposide, and ifosfamide (VIP) regimen, which has an efficacy comparable to that of BEP. Prior to the granulocyte colony stimulating factor (G‐CSF) era, the VIP regimen was associated with a higher incidence of hematological toxicity,^5^ but I think that the modern prophylactic use of pegylated G‐CSF in clinical practice has diminished this concern. Therefore, in metastatic GCC patients with intermediate or poor prognosis according to the IGCCCG classification, the replacement of bleomycin with ifosfamide might be considered as a means of avoiding adverse effects without reducing the treatment intensity when the risk of complications arises.

## Conflict of interest

The author declares no conflict of interest.
